# Intrinsic coagulation pathway-mediated thrombin generation in mouse whole blood

**DOI:** 10.3389/fcvm.2022.1008410

**Published:** 2022-11-28

**Authors:** Sandra Konrath, Reiner K. Mailer, Manu Beerens, Hanna Englert, Maike Frye, Piotr Kuta, Roger J. S. Preston, Coen Maas, Lynn M. Butler, Mark Roest, Bas de Laat, Thomas Renné

**Affiliations:** ^1^Institute of Clinical Chemistry and Laboratory Medicine, University Medical Center Hamburg-Eppendorf, Hamburg, Germany; ^2^Irish Centre for Vascular Biology, School of Pharmacy and Biomolecular Sciences, Royal College of Surgeons in Ireland, Dublin, Ireland; ^3^Central Diagnostic Laboratory, University Medical Center Utrecht, Utrecht University, Utrecht, Netherlands; ^4^Department of Clinical Medicine, The Arctic University of Norway, Tromsø, Norway; ^5^Clinical Chemistry and Blood Coagulation Research, Department of Molecular Medicine and Surgery, Karolinska Institute, Stockholm, Sweden; ^6^Department of Platelet Pathophysiology, Synapse Research Institute, Maastricht, Netherlands; ^7^Department of Functional Coagulation, Synapse Research Institute, Maastricht, Netherlands; ^8^Department of Data Analysis and Artificial Intelligence, Synapse Research Institute, Maastricht, Netherlands; ^9^Center for Thrombosis and Hemostasis (CTH), Johannes Gutenberg University Medical Center, Mainz, Germany

**Keywords:** thrombin generation, plasma contact system, factor XII, polyphosphate, CAT assay, diagnostics, coagulation, mouse whole blood

## Abstract

Calibrated Automated Thrombography (CAT) is a versatile and sensitive method for analyzing coagulation reactions culminating in thrombin generation (TG). Here, we present a CAT method for analyzing TG in murine whole blood by adapting the CAT assay used for measuring TG in human plasma. The diagnostically used artificial and physiologic factor XII (FXII) contact activators kaolin, ellagic acid and polyphosphate (polyP) stimulated TG in murine blood in a dose-dependent manner resulting in a gradual increase in endogenous thrombin potential and peak thrombin, with shortened lag times and times to peak. The activated FXII inhibitor rHA-Infestin-4 and direct oral anticoagulants (DOACs) interfered with TG triggered by kaolin, ellagic acid and polyP and TG was completely attenuated in blood of FXII- (*F12*^−/−^) and FXI-deficient (*F11*^−/−^) mice. Moreover, reconstitution of blood from *F12*^−/−^ mice with human FXII restored impaired contact-stimulated TG. HEK293 cell-purified polyP also initiated FXII-driven TG in mouse whole blood and addition of the selective inhibitor PPX_Δ12 ablated natural polyP-stimulated TG. In conclusion, the data provide a method for analysis of contact activation-mediated TG in murine whole blood. As the FXII-driven intrinsic pathway of coagulation has emerged as novel target for antithrombotic agents that are validated in mouse thrombosis and bleeding models, our novel assay could expedite therapeutic drug development.

## Introduction

The coagulation of blood culminates in the generation of thrombin, a protease that activates platelets and converts soluble fibrinogen to insoluble fibrin with subsequent polymerization and clot formation (1, 2). Kinetics of thrombin generation (TG) are highly complex and orchestrated by a delicate network of thrombin producing (procoagulant mechanisms) and terminating (anticoagulant mechanisms) reactions. Calibrated Automated Thrombography (CAT) allows for real-time monitoring of active thrombin concentrations in clotting plasma that reflect the complexity of generation and neutralization of the protease (3). CAT is based on the conversion of a thrombin-specific fluorogenic substrate, whose signal is normalized to an internal standard. This setup allows for highly sensitive monitoring of levels of the active enzyme in patient plasma samples in a diagnostic setting. The resulting thrombogram curve represents stimulus-dependent thrombin levels over time, providing information on (i) the time that elapses until TG is initiated (lag time), (ii) the time to maximal TG (time to peak), (iii) the maximal level of thrombin formed (peak thrombin), and (iv) the total amount of thrombin generated over time (endogenous thrombin potential, ETP, Supplementary Figure 1). The latter is often referred to as “area under the curve” and reflects the calculated time integral of thrombin concentrations in stimulated plasma.

TG involves complex activation of multiple clotting factors on membrane structures that expose phospholipids (PL), especially phosphatidylserine. As TG measurements are mainly performed with platelet-poor plasma (PPP) in the absence of blood circulating cells, addition of a synthetic PL mixture is required to provide a micelle-like surface structure that mediates coagulation factor surface recruitment and, by lateral diffusion, complex formation of these factors. In addition to the coagulation system that is analyzed by CAT in PPP, roles of platelets in TG can be studied using CAT analysis in platelet-rich plasma (PRP) (3). As such, PRP allows for analysis of the crosstalk between (activated) platelets and the coagulation system (primary and secondary hemostasis, respectively). However, the effect of other blood cells that have a major role in coagulation reactions *in vivo*, such as leukocytes and erythrocytes, on TG remains largely unidentified. Indeed, analysis of TG in whole blood samples is challenging as erythrocytes tend to sediment rapidly, leaving the ratio of fluid vs. cellular/membranous compartments undefined. Whole blood TG analysis can be further confounded by the release of hemoglobin from preanalytical hemolysis that may interfere with the fluorescent signal. As a result, TG assays developed for human whole blood are prone to high data variability (4), require extensive operating experience (5) and are unable to analyze more than one sample simultaneously (6, 7). These limitations can be circumvented by an experimental setup that enables continuous shaking of the blood sample throughout the TG measurement (8).

Since mouse models are an important tool for studying blood coagulation activation under physiological and pathological conditions, fluorogenic assays have also been developed to measure TG in mouse whole blood, where filter paper disks are typically used to prevent erythrocyte sedimentation. However, the usage of filter paper produced inaccurate readouts as the filter surface triggered contact activation of coagulation factor XII (FXII) (9, 10), which initiates TG. FXII is an essential part of the plasma contact system together with plasma prekallikrein (PK) and the non-enzymatic cofactor high molecular weight kininogen (HK). FXII circulates as a zymogen in blood and is activated by binding (“contact”) to negatively charged surfaces (11, 12), inducing a conformational change that leads to its conversion to the active protease FXIIa. Subsequently, FXIIa proteolytically cleaves PK forming activated plasma kallikrein (PKa), which in turn activates additional FXII zymogens in a positive feedback loop leading to amplified FXIIa production (fluid-phase activation). Sequential limited proteolysis of the FXIIa substrate factor XI (FXI), followed by factor IX and factor X, is referred to as the intrinsic pathway of coagulation. As a result, thrombin is produced followed by the formation of fibrin clots. FXII deficiency protects from pathological clot formation *in vivo* and genetic ablation of *F12* in mice (*F12*^−/−^) prevents arterial and venous thrombus formation without increasing the risk of bleeding (13, 14). Accordingly, *F12*^−/−^ mice are protected from thromboembolic disorders including ischemic stroke (15), cancer-associated thrombosis (16, 17), pulmonary embolism (18), and sepsis-mediated thrombosis (19).

Importantly, TG measurements in mouse whole blood have provided more detailed insights into the role of erythrocytes in FXII contact activation and its contribution to hyper- and hypo-coagulable states in experimental models. Here, we developed a modified CAT-based method (8) to precisely assess plasma contact system-triggered TG, in which samples were continuously mixed throughout the measurement to alleviate quenching of the fluorescence signal by red blood cells. Our data indicate that TG is largely reduced in the presence of direct oral anticoagulants (DOACs) and inhibitors targeting plasma contact system proteins, and is defective in whole blood from *F12*^−/−^ and FXI-deficient (*F11*^−/−^) mice. Therefore, our assay represents a promising new technique to study the impact of aberrant contact system protein activity on TG in mouse whole blood.

## Materials and methods

### Reagents

Kaolin was obtained from Sigma Aldrich. The activated partial thromboplastin time (aPTT) reagent Dade^®^ Actin^®^ FS containing ellagic acid and soybean phospholipids and Pathromtin SL^®^ were purchased from Siemens Healthcare Diagnostics. FXII contact activators STA^®^ - C.K. Prest^®^ and STA^®^ - Cephascreen^®^ were obtained from Diagnostica Stago (Asnières, France), while SynthAFax^®^, SynthASil^®^ and APTT SP reagent were purchased from IL Werfen (München, Germany). The following synthetic polyphosphates with different lengths were obtained from ICL Pharmaceuticals: ammonium polyphosphate (P42, #2 2846), ammonium polyphosphate (P30, #2 2840), sodium polyphosphate (P75, #7 9990), sodium polyphosphate (P70, #7 1480), sodium polyphosphate (P68, #7 1480). Dornase alfa, the recombinant human DNaseI (Pulmozyme^®^), was purchased from Roche. FXII purified from human plasma was obtained from Haematologic Technologies. The FXIIa-specific inhibitor rHA-Infestin-4 was supplied by CSL Behring. Thrombin generation assays were performed with the fluorogenic substrate Z-Gly-Gly-Arg-AMC, purchased from Bachem Bubendorf, Switzerland. The thrombin calibrator (α2-macroglobulin-thrombin complex, α2M-T) and MP reagent (containing phosphatidylserine, phosphatidylcholine and phosphatidylethanolamine at a ratio of 20:60:20) were obtained from Diagnostica Stago.

### Collection of mouse blood samples and plasma preparation

Wild-type (WT), FXI-deficient (*F11*^−/−^) and FXII-deficient (*F12*^−/−^) mice were anesthetized with isoflurane prior to blood sampling from the retro-orbital plexus. Orbital puncture was performed with a glass capillary 2 cm in length. Blood was collected into 0.5 ml tubes containing 3.2% trisodium citrate (9:1 blood to citrate ratio), while the first drop was discarded. At the time of blood sampling, mice were 12–40 weeks old. All animals were kept according to national guidelines for animal care at the University Medical Center Hamburg-Eppendorf.

Blood samples were used for TG measurements within the first 4 h after blood collection. Mouse platelet-poor plasma was prepared by centrifuging blood (2,500 × g for 15 min, twice). The procedure for isolation of erythrocytes and subsequent preparation of samples with different hematocrit levels was performed as described by Ninivaggi et al. (5).

### Real-time thrombin generation in mouse whole blood

The measurement of TG in mouse whole blood followed the protocol described by Wan et al. (8) with some major modifications. This assay was performed in 96-well round-bottom plates (Greiner Bio-One, #650101). Each well contained a final reaction mixture of 65 μl. First, 10 μl citrated mouse whole blood were added to the wells and then thoroughly mixed with 23 μl of the stimulator [kaolin (10 μg/ml), ellagic acid (0.2 μg/ml), polyphosphate (polyP, 2 μg/ml), aPTT reagents (final dilution of 1:20) or buffer (20 mM HEPES, 140 mM NaCl, 0.5% BSA, pH 7.35)]. The resulting mixture was immediately supplemented with 10 μl of the fluorogenic substrate Z-Gly-Gly-Arg-AMC (416 μM, reconstituted in 20 mM HEPES, 6% BSA, pH 7.35). Reaction mixtures were incubated for 10 min at 33 °C with orbital shaking. Finally, 22 μl CaCl_2_ (6.1 mM) were automatically added to each well with a dispenser of a Fluoroscan Ascent fluorometer (Thermo Scientific, Waltham, USA) and thrombin formation was analyzed with the CAT method using the Thrombinoscope software package (version 5.0.0.742). To ensure continuous and thorough shaking throughout the 30-min measurement period, TG in 36 wells was monitored at 6-s intervals in each experiment. If added, the concentration of exogenous phospholipids (MP reagent, Diagnostica Stago) was 4 μM. Each condition/blood sample was analyzed in triplicates and for calibration measurements the stimulator was replaced by an α2-macroglobulin-thrombin complex with known activity (Diagnostica Stago). In some experiments TG was measured in the presence of the FXIIa-specific inhibitor rHA-infestin-4 (Inf-4, 500 μg/ml), DOACs or the kallikrein-blocking reagent aprotinin (200 KIU/ml), which were incubated with blood samples for 30 min prior to stimulation. PolyP (7.1 and 10 μg/ml, respectively) isolated from human embryonic kidney (HEK)293 cells was used to trigger TG, whereas an exopolyphosphatase (PPX) variant that lacks the domains 1 and 2 (PPX_Δ12, 500 μg/ml) was absent or added to polyP 1 h before stimulation.

### Cell culture

HEK293 cells (ATCC: CRL1573) were maintained in Dulbecco's Modified Eagle Medium (DMEM) supplemented with GlutaMAX™ (Life Technologies, #10566016), 10% (v/v) heat-inactivated fetal calf serum (Life Technologies, #10082147), 100 U/ml penicillin and 100 μg/ml streptomycin (Life Technologies, #15140122). Cultures were incubated at 37 °C and 5% CO_2_.

### Extraction of polyP from HEK293 cells

Isolation of polyP was performed according to the procedure described by Nickel et al. (17), with minor modifications. In brief, confluent HEK293 cells were washed with PBS, detached from cell culture flasks by trypsinization, and centrifuged for 5 min at 200 × g. After washing the resulting cell pellet for three times with 50 mM Tris (pH 7.4), cells were incubated with sulfuric acid (0.3 M) and sodium chloride (3.5 M) for 5 min. The reaction mixture was adjusted to a pH of 7.4 using 2N sodium hydroxide followed by addition of 1 M Tris (pH 7.4) to achieve an osmolarity of 0.3 M. Cell lysates were first incubated with DNaseI (200 U/μl, in 3.5 mM MgCl_2_) for 30 min at 37°C and secondly with proteinase K (20 mg/ml) for 1 h at 37°C. After removal of cell debris by centrifugation (1,000 × g, 10 min), sodium iodide was added to the lysate at a final concentration of 4.5 M. The mixture was transferred to Qiagen PCR purification columns (Qiagen, #28106). Following two consecutive washing steps [with 10 mM Tris pH 7.4, 1 mM EDTA, 100 mM NaCl, 50% (v/v) EtOH], polyP was eluted in 50 mM Tris (pH 7.4) and stored at −80°C until usage.

### Analysis of polyP by urea-PAGE

Polyacrylamide gel electrophoresis (PAGE) with Tris/ boric acid/ EDTA (TBE) gels containing 15% (w/v) polyacrylamide and 7 M urea was performed to resolve polyP extracted from HEK293 cells. PolyP was visualized by negative staining with 4′,6-Diamidino-2-phenylindol (DAPI) as previously described (20). For staining, gels containing separated polyP were incubated with a fixative solution [25% (v/v) methanol, 5% (v/v) glycerol, pH 10.5] in the presence of DAPI (2 μg/ml) for 30 min with moderate shaking and without protection from ambient light. After washing the gels twice with fixative solution (without DAPI), the DAPI-bound polyP was photobleached using the ChemiDoc Imaging System (Bio-Rad). The images taken afterwards were analyzed using Image Lab software (Bio-Rad, version 6.1).

### Expression and purification of PPX_Δ12

The expression and purification procedure of recombinant PPX_Δ12 was performed as described previously (21). In brief, DH5α competent *E. coli* cells were cultured at 37°C on an orbital shaker after transformation with plasmid DNA (pTrcHisB backbone) coding for PPX_Δ12. Induction of protein expression through the *pTrc* promoter was achieved by addition of 0.5 mM isopropylthio-β-D-galactoside (Sigma-Aldrich). After lysis of cells by sonication, protein purification was performed using an ÄKTA™ start FPLC system (GE Healthcare) connected to a 1 ml HisTrap FF crude column (GE Healthcare). Histidine-tagged proteins were eluted with a buffer containing 20 mM NaH_2_PO_4_, 500 mM NaCl and 500 mM imidazole. The buffer of the elution fractions was changed to PBS, pH 7.4.

### Statistical analysis

Data were analyzed using GraphPad Prism 8.0 (GraphPad Software, San Diego, USA) and are shown as mean values ± standard deviation (SD). Depending on results of normality tests, more than two groups were compared using one-way ANOVA followed by Dunnett's multiple comparisons test (parametric statistics) or the Kruskal-Wallis test followed by Dunn's multiple comparison (non-parametric statistics). Value levels of statistical significance are considered as follows: *P* > 0.05 (ns – not significant), *P* < 0.05 (^*^), *P* < 0.01 (^**^), *P* < 0.001 (^***^).

## Results

### Establishment of a CAT assay in recalcified mouse whole blood

**Figure 1:**
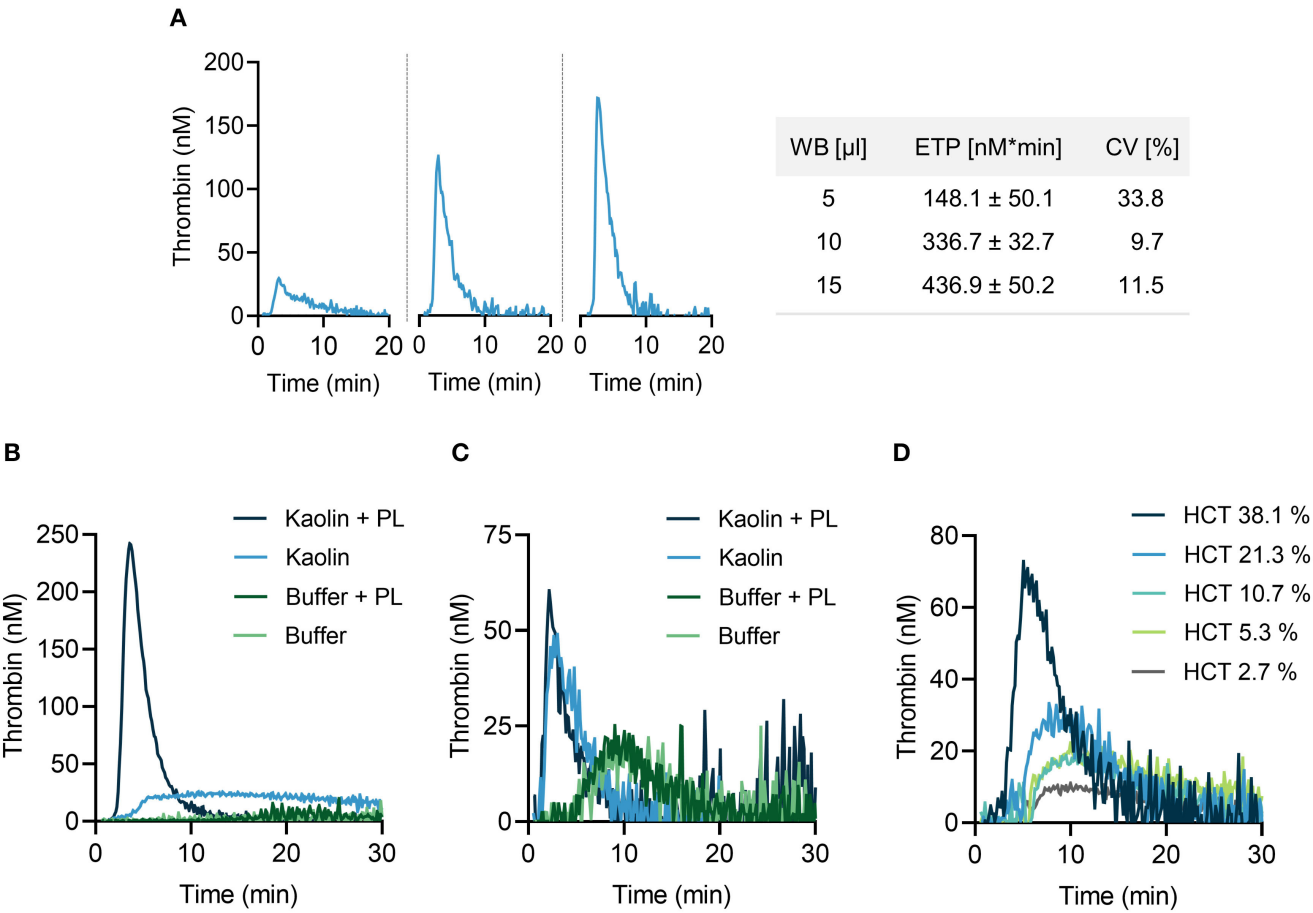
Thrombin generation (TG) in mouse whole blood assessed by CAT assay. **(A)** Left panel: real-time TG curves in 5 μl **(left)**, 10 μl **(middle)** or 15 μl **(right)** recalcified mouse citrate whole blood. Right panel: corresponding endogenous thrombin potential (ETP) values. Data are presented as mean ± standard deviation (SD) of 10 replicates and coefficient of variation (CV) was calculated thereof. **(B,C)** TG measurements performed in mouse PPP **(B)** and whole blood **(C)** in the presence or absence of phospholipids (PL, 4 μM). PPP and whole blood activated with buffer only served as control. **(D)** A fixed volume of mouse PPP was supplemented with increasing concentrations of erythrocytes as indicated by hematocrit levels (HCT, 2.7–38.1 %). In all experiments shown, TG was stimulated with kaolin (10 μg/ml). Representative TG curves measured in triplicates of *n* = 3 individual experiments.

We aimed to systematically optimize experimental conditions for CAT analysis in mouse citrate anticoagulated whole blood following addition of the contact activator kaolin (a white clay component) upon recalcification. Increasing the blood volume (from 5 to 15 μl in a total reaction volume of 65 μl) augmented TG activity, reflected by a shortening in lag time and time to peak, and largely increased peak thrombin and ETP (Figure 1A, left panels). Coefficient of variation (CV) of 10 replicates was lowest for 10 μl whole blood (9.7%) (Figure 1A, right panel), compared to 5 μl (33.8%) and 15 μl (11.5%), respectively. Changes in temperature (from 26 to 40°C) had minor effects on TG (data not shown). Thrombin formation proceeds on plasma membranes and addition of exogenous phospholipids (4 μM, containing phosphatidylserine, phosphatidylcholine and phosphatidylethanolamine at a ratio of 20:60:20) considerably enhanced kaolin-stimulated TG in mouse PPP (Figure 1B). TG was lower in whole blood as compared to PPP and addition of PL had no measurable effect on TG (Figure 1C). Plasma membranes of cells circulating in blood, e. g. platelets, provide the endogenous PL that promote FXIIa-driven TG (22). To assess the role of the hematocrit (HCT) in contact-initiated TG, mouse PPP was supplemented with increasing numbers of washed erythrocytes (Figure 1D). Increasing HCT levels dose-dependently increased kaolin-triggered TG, indicating a critical and unexpected procoagulant role of erythrocytes in TG (probably by their cell membranes).

For all further TG measurements, a reaction mixture containing 15% (v/v) mouse citrate-anticoagulated whole blood in 65 μl total reaction volume per well was used in the absence of exogenous PL at 33°C.

### Activation of TG in mouse whole blood by various contact activators

**Figure 2:**
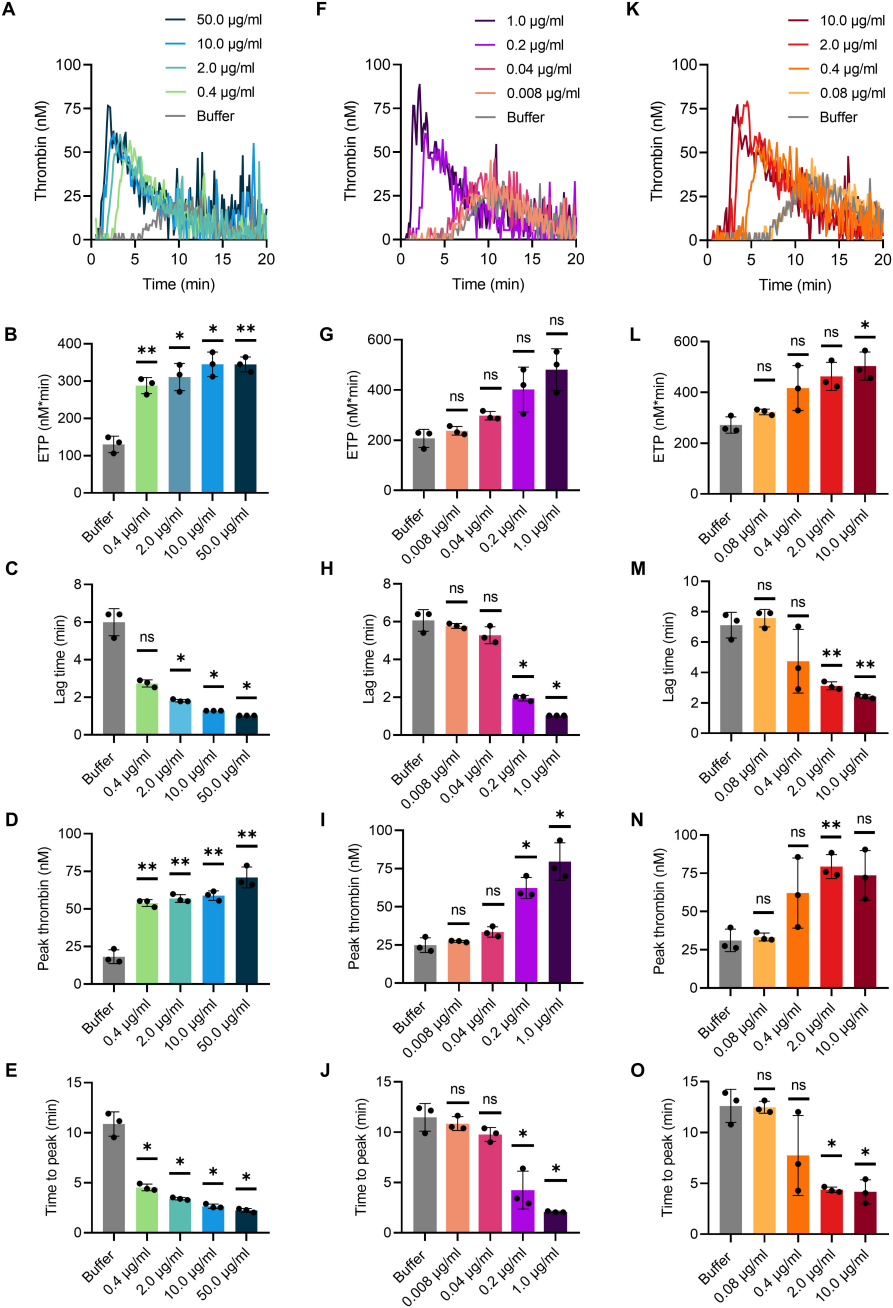
TG in mouse whole blood initiated by various contact activators. Citrate-anticoagulated venous whole blood collected from wild-type mice was supplemented in serial dilutions with **(A–E)** kaolin from 0.4 to 50.0 μg/ml, **(F–J)** ellagic acid from 0.008 to 1.0 μg/ml, and **(K–O)** synthetic polyP (polymer size: ≥400 mers) from 0.08 to 10.0 μg/ml in buffer. **(A–C)** Representative real-time TG graphs of *n* = 3 independent experiments measured in triplicates each and calculated endogenous thrombin potential [ETP; **(B,G,L)**], lag times **(C,H,M)**, maximal thrombin [peak height; **(D,I,N)**] and time to maximal thrombin [time to peak; **(E,J,O)**] are shown. Each condition was measured in triplicates. Bars represent mean ± SD. One-way ANOVA followed by Dunnett's multiple comparisons test was performed to assess statistical significance. *P* > 0.05 (ns, not significant), *P* < 0.05 (*), *P* < 0.01 (**).

**Figure 3:**
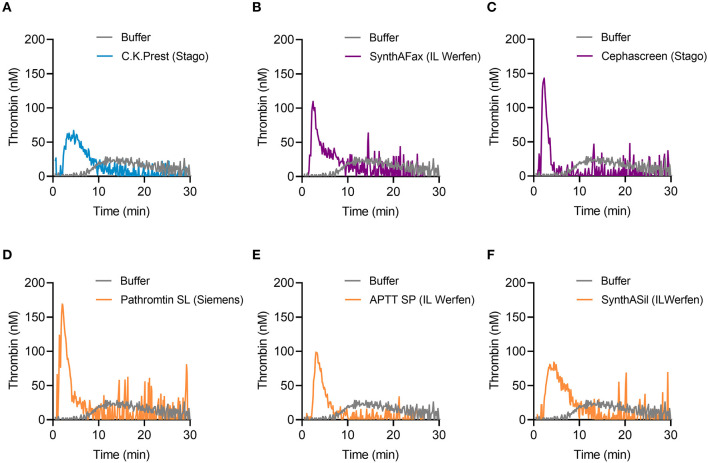
TG in mouse whole blood activated by commercially available aPTT reagents. **(A)** C.K.Prest (Stago), **(B)** SynthAFax (IL Werfen), **(C)** Cephascreen (Stago), **(D)** Pathromtin SL (Siemens), **(E)** APTT SP (IL Werfen), and **(F)** SynthASil (IL Werfen) were used with a final dilution of 1:20 each, to induce TG in mouse whole blood. Buffer serves as background control. Representative TG curves of *n* = 3 individual experiments are shown, while each condition was measured in triplicates.

**Figure 4:**
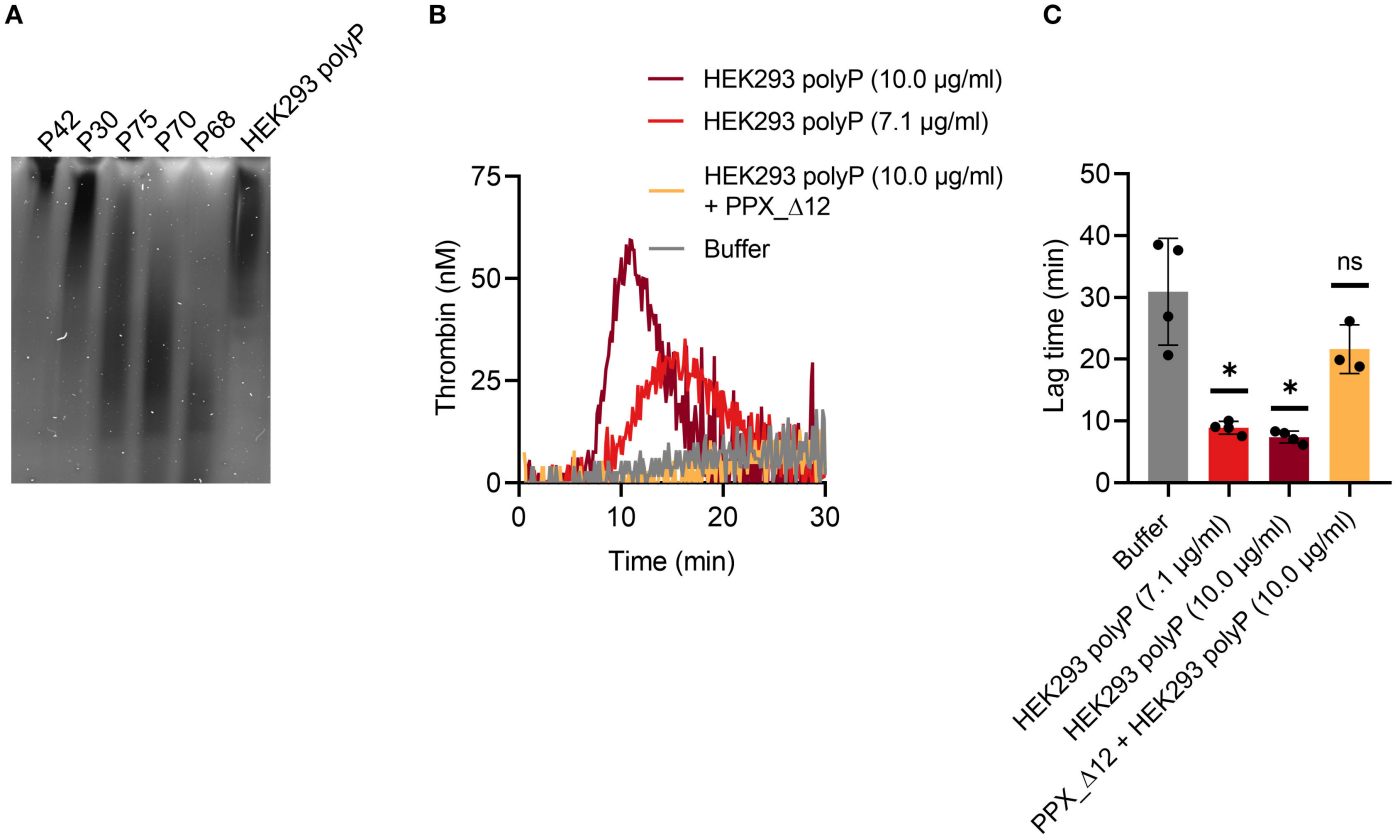
Cell-derived polyP triggers TG in mouse whole blood. **(A)** PolyP was isolated from 1.1 × 10^6^ HEK293 cells by anion exchange chromatography. Purified polyanions (50 ng/lane) were resolved by denaturating urea-PAGE [7 M urea/15% (w/v) polyacrylamide gels] and visualized using negative DAPI-staining. 20 ng/lane of synthetic polyP (P42–P68) was loaded as size and mass standard. Chain length of synthetic polyP - P 42: ≥ 650 mers, P30: ≥ 400 mers, P75: ≤ 650 mers, P70: ≤ 400 mers, P68: ≤ 150 mers. **(B)** Real-time TG in mouse whole blood was stimulated with cell-purified polyP (7.1 and 10 μg/ml, respectively) in presence or absence of recombinant polyP inhibitor PPX_Δ12 (500 μg/ml). Representative TG curves of *n* = 3 individual experiments measured in triplicates each. Corresponding time that elapses until TG is initiated (lag time) in **(B)** is shown in **(C)**. Mean ± SD are indicated in bars. Statistical analysis was performed using one-way ANOVA followed by Dunnett's multiple comparisons test. PolyP-stimulated TG was compared to buffer. *P* > 0.05 (ns, not significant), *P* < 0.05 (*).

TG in mouse whole blood was stimulated with different contact activators, including kaolin (Figures 2A–E), ellagic acid (Figures 2F–J) and synthetic long-chain polyP (≥400 mers; Figures 2K–O). All three contact activators dose-dependently increased total and maximum thrombin formation (peak thrombin), while reducing lag times and times to peak. Moreover, an array of commercially available contact activators from various manufacturers, commonly used in diagnostic coagulation laboratories to trigger aPTT assays, also effectively induced TG in mouse whole blood (Figures 3A–F). As synthetic polyP lacks calcium ions (Ca^2+^) that are bound to the polymer under physiological conditions and largely alter the structure and function of the polymer (18, 23), we next tested natural polyP of cellular origin for its capacity to trigger TG. We purified polyP from confluent HEK293 cells by an established anion affinity chromatography-based method (17). Eluted material was resolved by urea/PAGE and visualized by DAPI-negative staining. The dye bound to polyanions such as nucleotides or glycosaminoglycans does not photobleach and appears white, whereas polyP bound to DAPI photobleaches rapidly resulting in a black stain. This procedure identified purified HEK293 cell-derived polyP with a chain length dispersity of ~400 to >1,000 mers, similar to the synthetic polyP P30 (Figure 4A), and allowed for assessing the amount of extracted polymers by densitometry analysis. Cell-derived polyP (7.1 and 10 μg/ml, respectively) potently triggered TG in mouse whole blood (Figure 4B), as indicated by a significantly shortened lag time compared to buffer control (Figure 4C). Additionally, we preincubated our cell-purified polyP with an exopolyphosphatase (PPX) variant that lacks the domains 1 and 2 (PPX_Δ12) and binds specifically to polyP (but not to other polyanions) without degrading the polymers (21). Preincubation of anion exchange-purified polyP with PPX_Δ12 completely blocked polyP-induced TG, leading to lag times indistinguishable to buffer control, confirming that TG is exclusively driven by polyP.

### FXII dependency of contact activator-stimulated TG in mouse blood

**Figure 5:**
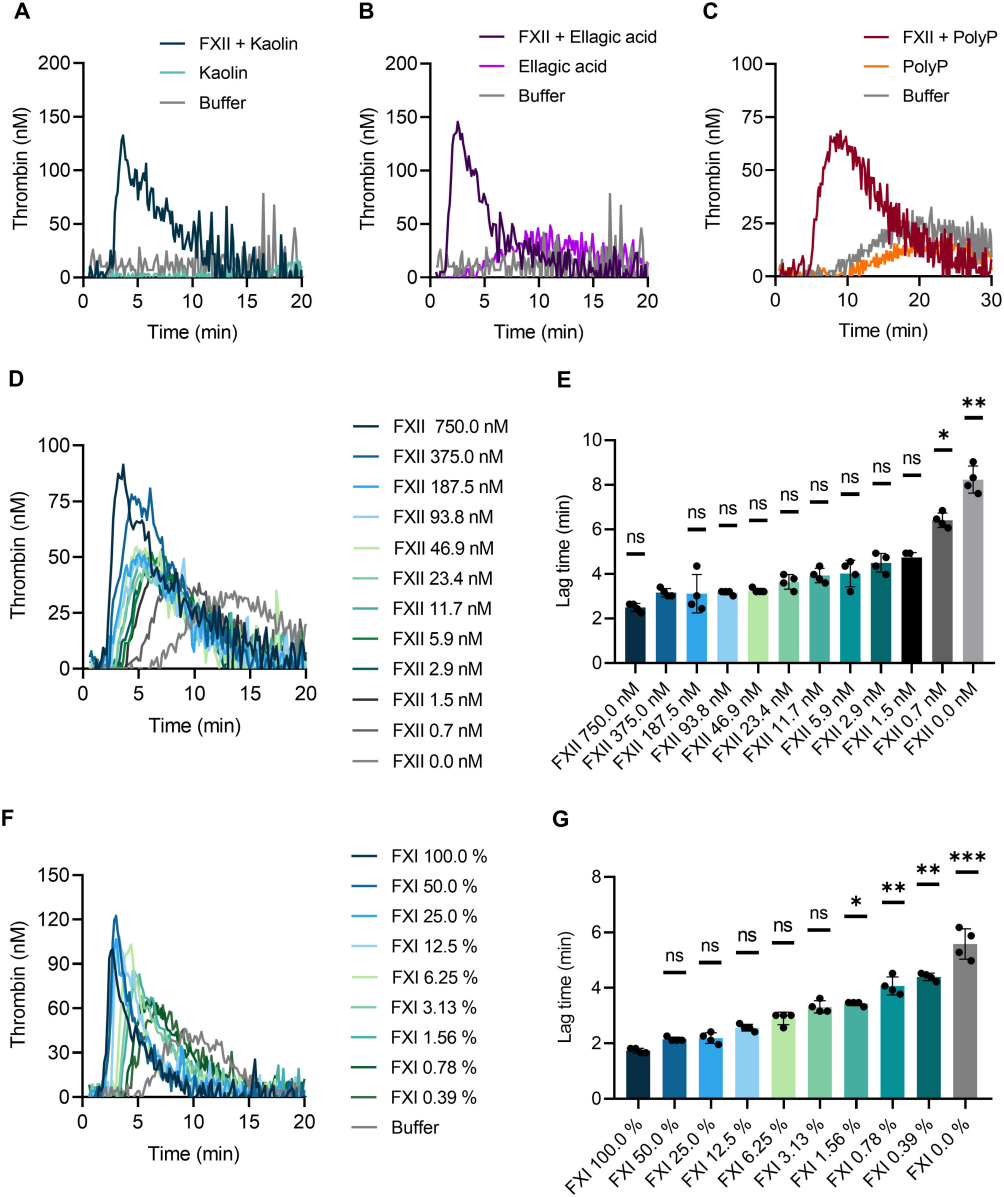
Contact-triggered TG is dependent on FXII and FXI levels in mouse whole blood. Venous whole blood collected from FXII-deficient (*F12*^−/−^) mice was stimulated with **(A)** kaolin (10 μg/ml), **(B)** ellagic acid (1 μg/ml), and **(C)** synthetic long-chain polyP (10 μg/ml, polymer size: ≥400 mers). **(A–C)** Real-time TG was assessed in recalcified blood following addition of human FXII (375 nM) or buffer. **(D,E)** Plasma-derived human FXII was added to whole blood collected from *F12*^−/−^ mice at concentrations ranging from 0 to 750 nM. **(F,G)** Defined mixtures of whole blood from wild-type and FXI-deficient (*F11*^−/−^) mice resulted in FXI levels of 100–0%. **(D–G)** Kaolin (10 μg/ml) stimulated TG. Corresponding times to onset of TG (lag times) in **(D,F)** are shown in **(E,G)**. Bars represent mean ± SD. Statistical significance was assessed by performing the Kruskal-Wallis test followed by Dunn's multiple comparison, with lag times compared to **(E)** 375 nM FXII or **(G)** 100% FXI. *P* > 0.05 (ns, not significant), *P* < 0.05 (*), *P* < 0.01 (**), *P* < 0.001 (***).

To confirm that TG is triggered by different negatively charged surfaces, we performed the CAT assay in whole blood of *F12*^−/−^ mice. Previously we have shown that FXII is not detectable in plasma of *F12*^−/−^mice by Western blotting (14). Kaolin (Figure 5A), ellagic acid (Figure 5B) and synthetic polyP (Figure 5C) completely failed to induce TG in *F12*^−/−^ mouse whole blood. Reconstitution of blood samples from *F12*^−/−^ mice with human plasma-derived FXII at physiological concentration (375 nM) fully restored defective TG upon stimulation with contact activators. Furthermore, we analyzed kaolin-induced TG in relation to FXII levels below and above the physiological concentration (Figure 5D). Supraphysiological FXII levels (750 nM) shortened the lag time, whereas reduced FXII concentrations (0–187.5 nM) prolonged lag times. The prolongation reached significant values at FXII <1.5 nM (Figure 5E). As pharmacological targeting of the FXII substrate FXI is gaining interest for safe interference with thromboembolism, we analyzed effects of altered FXI concentrations on TG in mouse whole blood. Lowering FXI levels dose-dependently reduced contact activation-triggered TG (Figure 5F) and prolonged lag times upon kaolin stimulation (Figure 5G).

**Figure 6:**
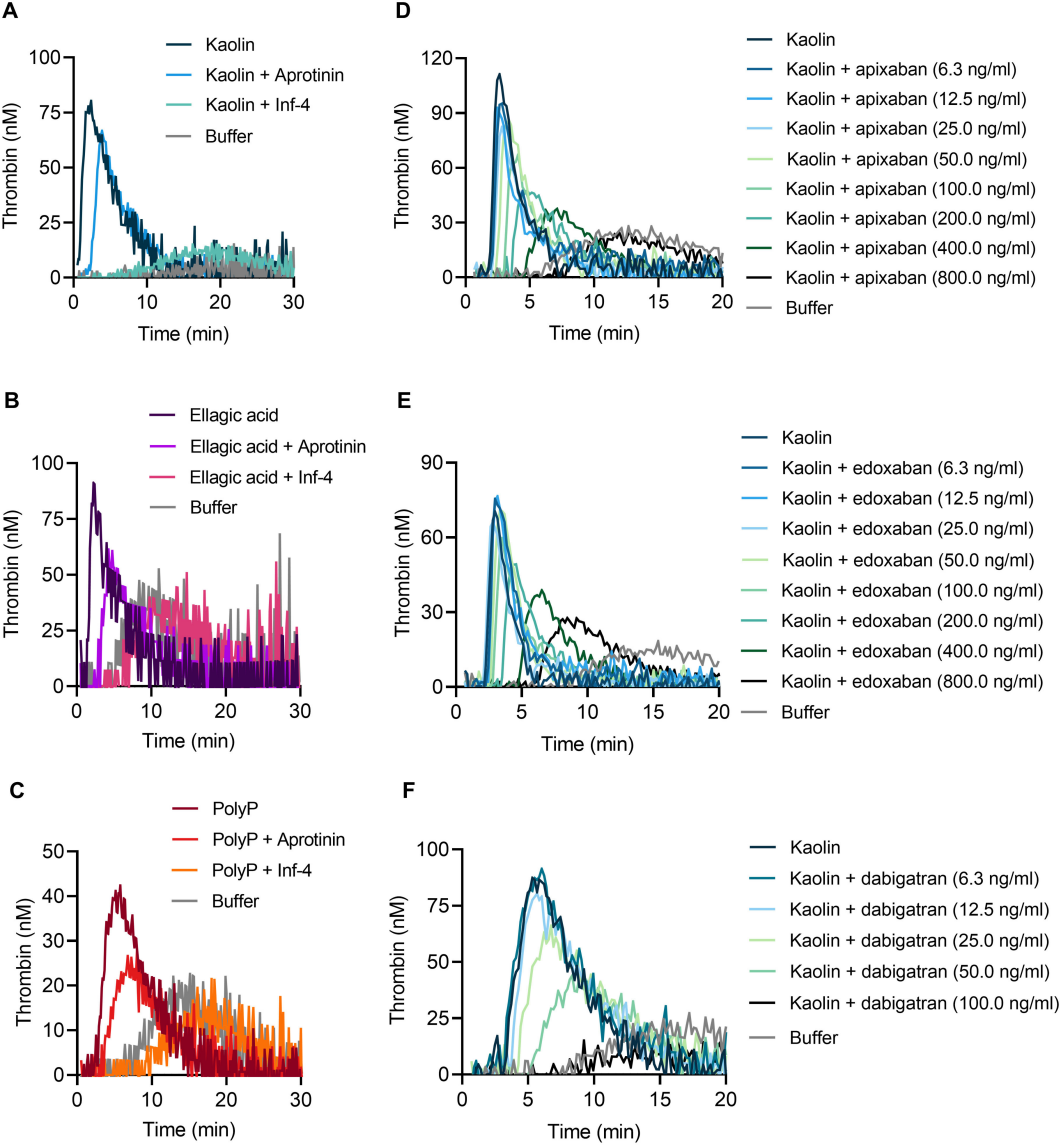
Targeting FXIIa, PKa, factor Xa, and thrombin reduces contact-triggered TG in murine whole blood. Representative thrombograms of citrate-anticoagulated whole blood obtained from WT mice stimulated with **(A,D–F)** kaolin (10 μg/ml), **(B)** ellagic acid (1 μg/ml), and **(C)** synthetic long-chain polyP (10 μg/ml, polymer size: ≥400 mers) in the presence or absence of **(A–C)** rHA-infestin-4 (Inf-4, 500 μg/ml) and aprotinin (200 KIU/ml) or direct oral anticoagulants (DOACs): **(D)** apixaban, **(E)** edoxaban, and **(F)** dabigatran. DOACs were added in serial dilutions from **(D,E)** 6.3 to 800 ng/ml or **(F)** 6.3 to 100 ng/ml. Buffer serves as background control. Representative curves of three individual experiments are shown. Each condition was measured in triplicates.

Consistent with impaired TG in FXII-deficient whole blood, addition of the specific FXIIa inhibitor rHA-infestin-4 [Inf-4, 500 μg/ml (24)] to blood of WT mice completely abolished TG induced by kaolin (Figure 6A), ellagic acid (Figure 6B) and polyP (Figure 6C). FXIIa is produced by zymogen binding to negatively charged surfaces or proteolytic conversion of FXII by PKa. Importantly, blocking PKa by aprotinin (200 KIU/ml) reduced kaolin, ellagic acid and polyP-triggered TG (Figures 6A–C). These data are consistent with the notion that TG in mouse whole blood is critically dependent on FXII contact activation, while contact activation-formed FXIIa is amplified by PKa-mediated FXII fluid phase activation. To evaluate our assay for analysis of currently used anticoagulants, we spiked mouse whole blood with DOACs. The presence of the factor Xa inhibitors apixaban or edoxaban blocked contact activation-triggered TG in a dose-dependent manner (Figures 6D,E). In a proof-of-concept experiment, the direct thrombin inhibitor dabigatran completely abolished kaolin-induced TG at a dose of 100 ng/ml (Figure 6F).

## Discussion

Thromboembolic and bleeding diseases represent a significant medical burden. Coagulation diagnostics are required to assess risk of thrombosis or bleeding, monitor anticoagulation or substitution therapy, and provide a standard of care for patients with coagulation abnormalities. Coagulation diagnostics include analysis of plasma (e.g., activity of factor VIII), platelets (e.g., ADP release) and recently whole blood. The latter allows to assess plasma coagulation, procoagulant activity of circulating cells including platelets and leukocytes, and the intimate crosstalk thereof. In addition to platelets, the presence or absence of leukocytes such as monocytes and neutrophils plays a major role in secondary haemostasis, e.g., by exposing tissue factor (TF) (25), initiating the extrinsic coagulation pathway. Activated eosinophils, macrophages, and most especially neutrophils release anionic extracellular DNA into the extracellular space (neutrophil extracellular traps, NETs), resulting in FXII activation and degradation of tissue factor pathway inhibitor (TFPI) (26–28).

CAT-based TG in human PPP and PRP is a sensitive method for monitoring formation and neutralization of the main executor of coagulation, thrombin. Tchaikovski et al. adapted the human CAT assay for mouse PPP and showed that mouse plasma appears to have higher procoagulant activity as compared to human samples, characterized by very short lag times (29). In fact, monitoring TG from its initial phase and calculating TG parameters was challenging. To overcome this limitation, the authors analyzed TG at low temperature of 33°C, which caused the procoagulant reactions to slow down and prolonged lag times. We adapted this strategy for our study, noting that performing our CAT assay at different temperatures had only minor effects on TG profiles.

Monitoring TG in human and mouse whole blood required modifications of existing protocols as erythrocytes rapidly sediment, quench fluorescence signals and contribute to clot contraction during TG measurements. Whole blood TG assays are further limited by high intra-assay variation (4), handling issues (5) or the incapacity to analyze multiple samples simultaneously (6, 7). Recently, a fluorogenic TG assay in human whole blood with constant sample mixing by permanently moving the microtiter plate led to highly reproducible data (8). Furthermore, established TG analysis of mouse whole blood used filter paper disks to minimize erythrocyte sedimentation by restraining them in a matrix (10). However, filter papers function as contact activators leading to significant preanalytical FXIIa formation, limiting the analysis of the intrinsic pathway of coagulation. Here, we developed a novel assay that allows to reliably measure TG triggered by contact activation in mouse whole blood in the absence of filter papers, while data variability between replicates (Figure 1) was comparable to the filter paper-based TG technique presented by Ninivaggi and colleagues (10). In contrast to previous TG assays (29), we used about half of the blood sample volume allowing for multiple analyses from minimal blood volumes.

To stimulate TG in mouse whole blood in a dose-dependent manner in our assay (Figure 2), we used the polyanions kaolin, ellagic acid and synthetic polyP (Figure 2), which are known to activate FXII (30–32). Of note, the chain length of synthetic polyP correlates with its procoagulant potential in plasma *ex vivo* (32), whereas natural polyP is complexed with Ca^2+^ and forms insoluble nanoparticles challenging a role for polyP chain length for coagulation *in vivo* (23). Ca^2+^-polyP microparticles induce FXII activation on procoagulant platelet surfaces (18, 23), various cancer cells, and extracellular tumor-derived vesicles such as prostasomes, and drive pulmonary embolism in mouse models (16, 17). Because of this critical role in triggering thromboembolic events in animals, we also tested the potential of natural polyP isolated from HEK293 cells in stimulating TG in mouse whole blood. Methods for isolation of polyP from cells and cell-derived components are either based on phenol/chloroform extraction (33) or anion exchange chromatography (34, 35). While the former method extracts predominantly water-soluble (short-chain) polyP, preparation by anion exchange resin contains both soluble (short-chain) and insoluble (long-chain) polyP. Indeed, we isolated long-chain polyP (~400 to >1000 mers) from confluent HEK293 cells using commercially available ion exchange resins (Figure 4). Similarly, synthetic polyP (Figure 2), cell-derived polyP triggered TG in mouse whole blood (Figure 4). PolyP extracted with anion exchangers used for DNA purification has been claimed to be contaminated with procoagulant silica that presumably had leaked from the column matrix (36). However, targeting cell-derived polyP with recombinant PPX_Δ12 that binds specifically to polyP of chain length >35 but not to other polyanions (21), completely inhibited TG. These data indicate that silica, if present at all, has minor importance in TG induced by anion exchanger-purified polyP using our method.

In addition to polyanions, extracellular vesicles (EV) derived from platelets and erythrocytes have the potential to stimulate TG (37). Erythrocyte-derived EV contribute to intrinsic pathway-mediated TG by two mechanisms: (i) contact activation of FXII and (ii) a FXII-independent pathway driven via PK activation and subsequent stimulation of factor IX (FIX) (38). Consistent with this study, PKa may activate FIX *in vitro* (39). FXII deficiency or blocking FXIIa by Inf-4, but not PK inhibition with aprotinin (Figure 4), completely abrogated TG in mouse whole blood exposed to polyanionic surfaces. These data support the notion that TG was initiated through classical FXII contact activation, but not via a FXII-independent mechanism. The discrepancy may reflect the fact that FXII-independent procoagulant mechanisms result from long-term storage of erythrocytes (38).

Our novel CAT assay requires further analysis, for example, in animal models with subtle and diffuse coagulation disturbances, including hypofibrinolysis in acute sepsis and traumatic injury. In severe sepsis, widespread activation of the coagulation system involves rapid “consumption” of several clotting factors with persistent thrombus formation. This leads to a hypocoagulable state (40) termed disseminated intravascular coagulation (DIC) that is associated with multiorgan failure and high mortality. Sepsis-mediated DIC is characterized by excessive formation of thrombin in which pathogen-derived polyP activates FXII (41). Clinically, DIC resembles trauma-induced coagulopathy (TIC), where patients initially tend to bleed but later change to a more hypercoagulable state associated with venous thromboembolism and multiorgan failure (42). Our CAT assay in mouse whole blood could embed TG in a more physiologic setting and offer additional insight into these complex coagulopathies.

In conclusion, we established a method to specifically assess TG, driven via the plasma contact system in mouse whole blood. Since different tested polyanions triggered FXIIa-mediated TG, our method provides a useful tool to identify other, yet unknown candidates contributing to TG through contact activation. As FXII deficiency or FXIIa inhibition led to defective TG in mouse whole blood, our technique may be of interest for the development of antithrombotic drugs, as FXII inhibitors interfere with thrombosis while sparing hemostasis (43, 44).

## Data availability statement

The original contributions presented in the study are included in the article/Supplementary material, further inquiries can be directed to the corresponding author/s.

## Author contributions

SK performed the experiments and analyzed the data of this study. RM, HE, MB, MF, PK, RP, CM, LB, MR, and BL provided critical tools, discussed data, and critically read the manuscript. SK and TR wrote and edited the manuscript. All authors contributed to the article and approved the submitted version.

## Funding

This research was supported by the Rudolf-Marx-Research-Grant of the Society of Thrombosis and Haemostasis Research e.V. (GTH) and the German Research Foundation (DFG, project number 470698011 to RM). TR acknowledges the German Research Foundation (grants A11/SFB 877, P6/KFO 306, and B8/SFB 841) for funding.

## Conflict of interest

Authors BL and MR are employees of Synapse Research Institute, part of Diagnostica Stago. The remaining authors declare that the research was conducted in the absence of any commercial or financial relationships that could be construed as a potential conflict of interest.

## Publisher's note

All claims expressed in this article are solely those of the authors and do not necessarily represent those of their affiliated organizations, or those of the publisher, the editors and the reviewers. Any product that may be evaluated in this article, or claim that may be made by its manufacturer, is not guaranteed or endorsed by the publisher.

## Abbreviations

CAT: Calibrated Automated Thrombography
ETP: endogenous thrombin potential
TG: thrombin generation
Inf-4: recombinant human albumin-tagged (rHA)-Infestin-4
PK: plasma prekallikrein
PL: phospholipids.
