# Supplementary material for: Intrinsic coagulation pathway-mediated thrombin generation in mouse whole blood
Source: Front Cardiovasc Med. 2022 Nov 28;9:1008410. doi: 10.3389/fcvm.2022.1008410 (PMC9742269; doi:10.3389/fcvm.2022.1008410)
Supplement: Supplementary file 1 [file Data_Sheet_1.docx]

**Supplementary material**

**Figure S1. Thrombin generation (TG) curve and its related parameters.** The lag time represents the elapsed time until TG is initiated. The elapsed time to maximal TG is given by the time to peak. Maximum thrombin formed is indicated by peak thrombin and the area under the curve corresponds to the total amount of thrombin generated (endogenous thrombin potential, ETP).
